# Augmented Reality Navigation System Enhances the Accuracy of Spinal Surgery Pedicle Screw Placement: A Randomized, Multicenter, Parallel‐Controlled Clinical Trial

**DOI:** 10.1111/os.14295

**Published:** 2025-01-15

**Authors:** Yichao Ma, Jiangpeng Wu, Yanlong Dong, Hongmei Tang, Xiaojun Ma

**Affiliations:** ^1^ Shanghai University of Traditional Chinese Medicine Shanghai China; ^2^ Department of Orthopedics, Shanghai General Hospital Shanghai Jiao Tong University School of Medicine Shanghai China; ^3^ Linyan Medical Technology Company Limited Shanghai China

**Keywords:** accuracy, augmented reality, navigation, pedicle screw, thoracolumbar spine

## Abstract

**Objective:**

The pedicle screw insertion technique has evolved significantly, and despite the challenges of precise placement, advancements like AR‐based surgical navigation systems now offer enhanced accuracy and safety in spinal surgery by integrating real‐time, high‐resolution imaging with virtual models to aid surgeons. This study aims to evaluate the differences in accuracy between novel AR‐guided pedicle screw insertion and conventional surgery techniques.

**Methods:**

A randomized controlled trial was conducted from March 2019 to December 2023 to compare the efficacy of AR‐guided pedicle screw fixation with conventional freehand surgery using CT guidance. The study included 150 patients, aged 18–75, with 75 patients in each group. The total number of pedicle screws planned for the clinical trial placement was 351 and 348 in the experimental and control groups. The safety and efficacy of the procedures were evaluated by assessing screw placement accuracy and complication rates.

**Results:**

In the full analysis set (FAS) analysis, the difference in the excellent and good rates of screw placement (experimental group − control group) and 95% confidence interval was 6.3% [3.0%–9.8%], with a *p* value of 0.0003 for the superiority test. In the FAS sensitivity analysis, the success rate was 98.0% (344 out of 351) in the experimental group and 91.7% (319 out of 348) in the control group, with a difference and 95% confidence interval of 6.3% [2.9% and 9.8%, respectively]. In the per‐protocol set (PPS) analysis, the difference in the excellent and good rates of screw placement between the experimental and control groups, and the 95% confidence interval was 6.4% [3.3%–9.5%], with a *p* value of 0.0001 for the superiority test. In the actual treatment set (ATS) analysis, the excellent and good rates of screw placement were 99.1% in the experimental group and 91.7% in the control group. The difference in the excellent and good rates of screw placement (experimental group − control group) and 95% confidence interval was 7.3% [4.1%–10.6%], with a *p* value of < 0.0001 for the superiority test.

**Conclusions:**

The AR surgical navigation system can improve the accuracy of pedicle screw implantation and provide precise guidance for surgeons during pedicle screw insertion.

## Graphical abstract

We evaluate the difference between a novel AR‐guided pedicle screw insertion and conventional surgery. Results show that the AR surgical navigation system can enhance the accuracy of pedicle screw implantation and offer precise guidance to surgeons during the insertion process.

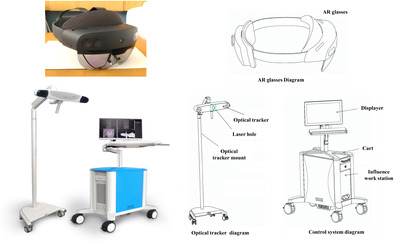

## 1 Introduction

The pedicle screw insertion technique was first reported by Boucher [1] in 1959, and has undergone further development in subsequent years. Since then, its superior stabilizing effects in various regions of the spine have been robustly demonstrated by clinical and biomechanical studies. Currently, pedicle screw insertion is widely employed in three‐column spinal fixation, having become the most commonly used technique in the thoracolumbar spine [2, 3]. However, serious nerve, vascular, and visceral injuries can occur due to screw misplacement; accurate placement of pedicle screws is crucial to prevent intraoperative nerve and vascular damage [4]. Therefore, accuracy in placement is paramount and key to surgical success.

Various techniques have been employed to ensure precise pedicle screw placement, including freehand, image‐guided surgery, that is, intraoperative X‐ray or computed tomography (CT) navigation technology and robotic guidance, and three‐dimensional (3D) printed navigation guidance templates [5, 6, 7, 8, 9]. However, despite the emergence of advanced technologies, pedicle screw placement remains technically demanding. The accuracy of screw placement varies depending on various factors, ranging from 28% to 100% [10, 11].

Augmented reality (AR) is an emerging branch of virtual reality (VR) technology. AR technology integrates computer‐generated virtual information into real‐world scenes and superimposes virtual scenes, models, or system information onto real scenes. This creates an interactive platform between the real world, the virtual world, and users, enhancing the user experience and embodying the concept of enhancing reality. In recent years, AR technology has developed rapidly in various fields. In the field of orthopedics, AR technology has shown broad application prospects, including in preoperative planning, surgical procedures, technical training, rehabilitation training, and self‐learning; it also brings substantial economic and social benefits [12, 13, 14, 15, 16]. In spinal surgery, due to the complexity of spinal structure and function, along with the diversity of spinal diseases, surgical risks and challenges remain high. The continuous development and maturity of AR technology have created favorable conditions for planning spinal surgery, simulating surgical approaches, improving surgical outcomes, reducing surgical risks, and training young orthopedic surgeons [17, 18, 19, 20, 21].

Therefore, our surgical navigation system, developed and produced based on AR technology, processes, analyses, integrates, and outputs the preoperative imaging data of patients to ensure the reconstruction of a 3D image that is highly consistent with the actual anatomical structure during surgery. Through registration techniques, virtual and real object coordinates were precisely matched and presented to the surgeon. The images are characterized by high clarity, resolution, structured illustration, and strong stereoscopic effect, assisting surgeons in the real‐time, rapid, and accurate identification of anatomical structures and their spatial relationships during surgery, thus facilitating precise surgical operations. The purpose of this study is to (i) evaluate the accuracy of pedicle screw placement, (ii) assess the safety of the procedure, and (iii) determine the overall efficacy of AR‐guided pedicle screw fixation compared to conventional methods.

## 2 Materials and Methods

### 2.1 Ethics Statement

The clinical trial protocol was approved by the Ethics Committees of Shanghai General Hospital affiliated with Shanghai Jiao Tong University, Changzheng Hospital affiliated with the Second Military Medical University, and Pudong New Area People's Hospital in Shanghai. The approval number is Institutional Review Board (IRB): YL001, Independent Ethics Committee (IEC) IRB2021‐039, and the registration no. of clinical trial is ChiCTR1900021878. All patients participating in this study provided written informed consent prior to their inclusion, in accordance with the ethical guidelines. We conducted a clinical randomized controlled trial in order to evaluate the safety and efficacy of the AR surgical navigation system in assisting pedicle screw placement during spinal fixation surgery. This trial employed a randomized controlled design, with CT‐guided conventional manual surgery as the control, to assess the safety and efficacy of the AR surgical navigation system in assisting pedicle screw fixation during spinal surgery.

### 2.2 Patient Recruitment

Inclusion criteria were as follows:

1. Age between 18 and 75 years.
2. Patients who underwent initial posterior spinal fusion surgery and voluntarily participated in the clinical trial and signed an informed consent form.
3. Surgical segments include two or three vertebral bodies from T1 to S1, requiring pedicle screw placement, and the treatment plan allows for open surgery.
4. CT images capable of 3D modeling are available at the hospital, either from CT images taken on admission or meeting the modeling requirements.

Exclusion criteria were as follows:

1. Pregnant or lactating women.
2. Patients with systemic diseases such as severe bleeding disorders, severe heart disease, severe respiratory disease, or those unable to tolerate anesthesia or surgery.
3. Patients unable to tolerate the positioning requirements of spinal surgery.

All surgeries were performed by two specialist surgeons with 10–20 years of experience in spinal fixation. During the clinical trial, treatments were strictly conducted according to equipment specifications and protocols in order to avoid operational biases between researchers and across surgeries. To prevent center bias, researchers received sufficient training to become familiar with the product operation procedures, ensuring their equivalent technical proficiency.

### 2.3 Study Design

This trial employed a randomized, single‐blind design; the subjects will be randomly stratified according to the planned number of screws to be inserted. Prior to surgery, participants were assigned to either the experimental or control group based on random numbers generated by statistical software. To minimize subjective bias, a third‐party evaluator involved in imaging assessments will not participate in the surgeries or have access to the medical records of the study, ensuring that they remain unaware of the subjects' assigned groups.

Analysis of the primary efficacy endpoints will be conducted simultaneously based on the full analysis set (FAS) and the per‐protocol set (PPS). Furthermore, all baseline demographic data and secondary efficacy analyses will be conducted based on the FAS, while safety assessment will also be based on the FAS; thus, SS is not separately defined. The experimental group will use the AR surgical navigation system to collect data from patients undergoing radical surgery with the Holonavi S model from March 2019 to December 2023, in the hospital. The control group will undergo conventional manual operations guided by CT‐guided, with data being collected from patients undergoing open surgery in the hospital from March 2019 to December 2023.

The sample size ratio between the experimental and control groups was 1:1. The sample size was determined on the basis of the research hypothesis and the estimated level of expected efficacy. The total number of patients enrolled in this study was 150. The trial employed a randomized, single‐blind design. Participants were stratified and randomly assigned to either the experimental group or the control group based on the number of screws to be implanted, using random numbers allocated by statistical software before the procedure. Since participants had no visual contact with the instruments, a single‐blind design was feasible. Statistical analysis will be conducted based on the aforementioned population; clear definitions of the analysis population will be established before the start of statistical analysis. The analysis population in this study includes the FAS, which comprises a data set consisting of all randomized subjects who received the investigational product, following the principle of intention‐to‐treat (ITT). For patients who did not observe the primary efficacy endpoint, the worst‐case carry forward (WCCF) strategy was proposed to impute missing data. The term PPS refers to the subset of the treated population who completed the trial and excluded serious protocol deviations such as violations of inclusion/exclusion criteria.

The analysis of the primary efficacy endpoint will be conducted simultaneously on both the FAS and the PPS. In this study, the primary evaluation indicator was the success rate of pedicle screw placement. A comparison of the success rates between the experimental and control groups will be conducted in order to demonstrate that the investigational product meets clinical requirements. If the 95% confidence interval for the difference in pedicle screw placement success rates between the experimental and control groups falls entirely in the positive region, that is, the lower limit of the two‐sided 95% confidence interval is greater than 0, it can be concluded that the hypothesis of superiority is established. This indicates that the investigational product meets the clinical requirements.

The process of analyzing the data set is outlined below.

1. No use of study devices; participants were assigned random numbers but received no treatment related to the study, including both investigational and control products.
2. Violation of inclusion/exclusion criteria; participants did not meet the inclusion criteria outlined in the protocol or met the exclusion criteria specified in the protocol. This violation of inclusion/exclusion criteria significantly affected the results of the primary efficacy endpoint.
3. Failure to achieve the primary endpoint: Participants were unable to attain the primary endpoint, which is the excellent rate of pedicle screw placement. Refer to the criteria for assessing the primary endpoint in detail.
4. Cross‐over enrollment: Participants randomized to the experimental group received the control group product, whereas participants randomized to the control group received the experimental group product.
5. FAS = number of subjects enrolled in the trial − number of subjects who received no study devices. PPS = FAS − number of subjects with serious protocol violations; ATS = PPS + number of subjects with only cross‐group assignments. If there are instances of cross‐group assignments in the trial, ATS will be displayed; if there are no instances of cross‐group assignments, ATS will not be displayed. The number of subjects with serious protocol violations = number of subjects violating the inclusion/exclusion criteria + number of subjects who did not achieve the primary endpoint + number of subjects with cross‐group assignments.

### 2.4 AR Navigation System

**FIGURE 1:**
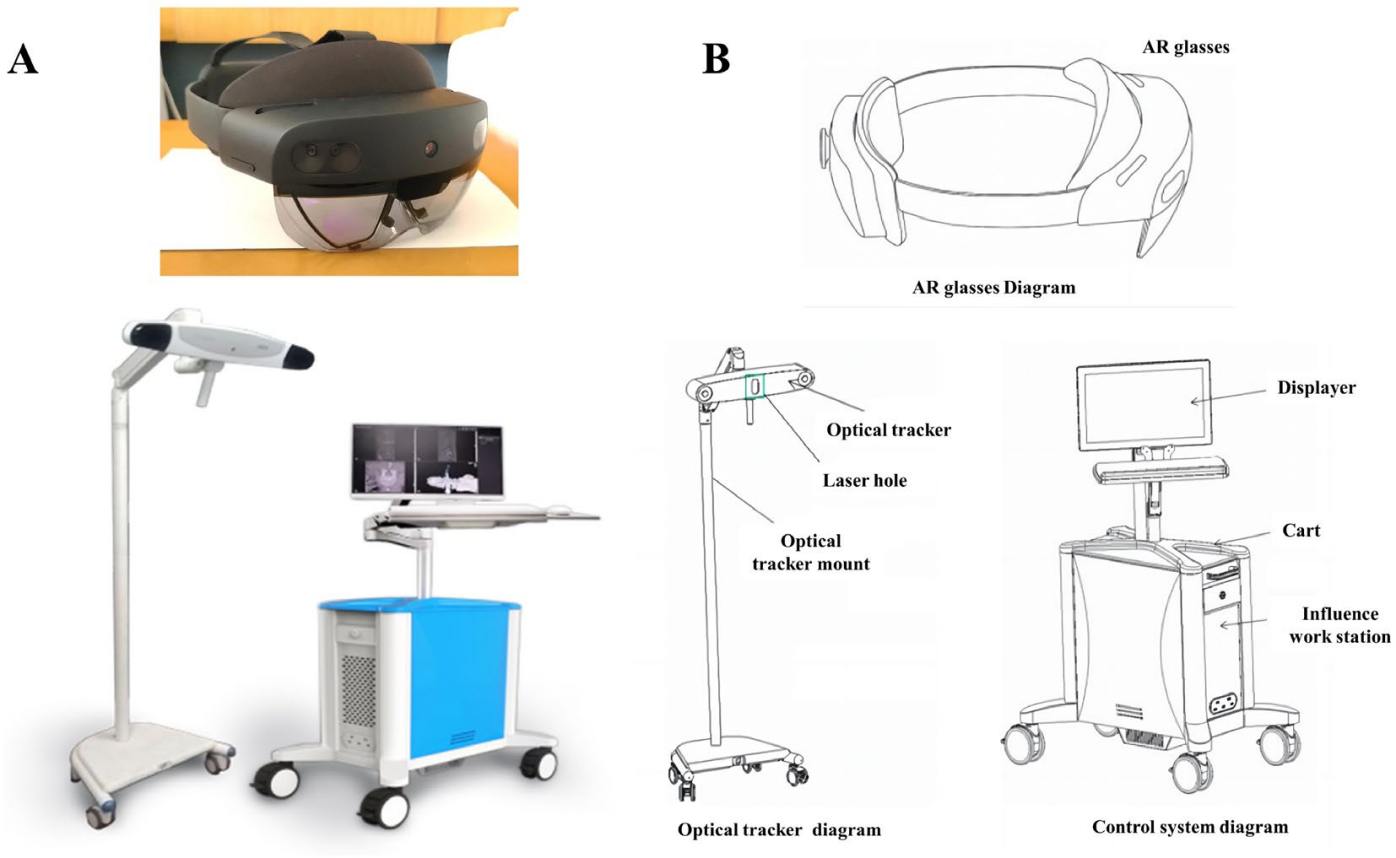
Diagram of the AR navigation system. (A) Photo of the AR navigation system. (B) Functional structure diagram of AR navigation.

A high‐performance graphic workstation with an AR navigation system was developed by the Shanghai Linyan Medical Technology Co. Ltd. (located at 528 Ruiqing Road, Pudong New Area, Shanghai, China). The diagram of the AR navigation system is shown in Figure 1. The hardware architecture consists of a high‐performance graphical workstation (Holonavi, China), an optical transparent head‐mounted display (HMD) (HoloLens, Microsoft, USA), and an optical tracking system (NDI, Polaris Vega, Canada). The control system, optical tracking, AR‐specific glasses, and navigation software are non‐contact components for patients and can be used in the operating room environment.

The control system comprises a monitor, cart, and image workstation designed for compatibility with standard mouse and keyboard usage; its primary functions include hosting AR surgical navigation system software, storing data, and providing technical support. The monitor displays the images and provides information, the mouse and keyboard were used to interact with the control system and prepare for navigation system usage, and the optical tracking system includes an optical tracker and optical tracker stand. Its primary functions include emitting infrared light, capturing reflected infrared light, converting optical information into digital data, and providing positioning functionality in order to achieve 2D‐3D registration between preoperative CT and intraoperative C‐arm images for navigation purposes. The HMD with high‐resolution micro displays (1280 × 1024 24‐bit color pixels per eye), connects to the workstation, and provides surgeons with real‐time overlay of virtual images onto the real world.

## 3 Measurements

### 3.1 Primary Evaluation Criteria

The main evaluation index of this clinical trial was the excellent rate of pedicle screw placement. Combining literature, clinical practice experience, and expert consensus, we defined the excellent rate as follows: the proportion of pedicle screws that met the excellent judgment criteria among all evaluated pedicle screws [22, 23, 24]. This includes pedicle screws that are completely within the pedicle without penetrating the cortical bone of the pedicle or those that penetrate the cortical bone of the pedicle with a penetration distance of less than 2 mm.

The detailed evaluation criteria for pedicle screw placement are as follows:

A: Fully intrapedicular position without breach of the pedicle cortex.

B: Exceeding the pedicle cortex < 2 mm.

C: Exceeding the pedicle cortex 2–4 mm.

D: Exceeding the pedicle cortex 4–6 mm.

E: Exceeding the pedicle cortex > 6 mm or is outside of the pedicle.

Grades A and B can be considered satisfactory operation results. In Grades C to E, neurological symptoms may occur and can be evaluated as an unsatisfactory surgical result.

**FIGURE 2:**
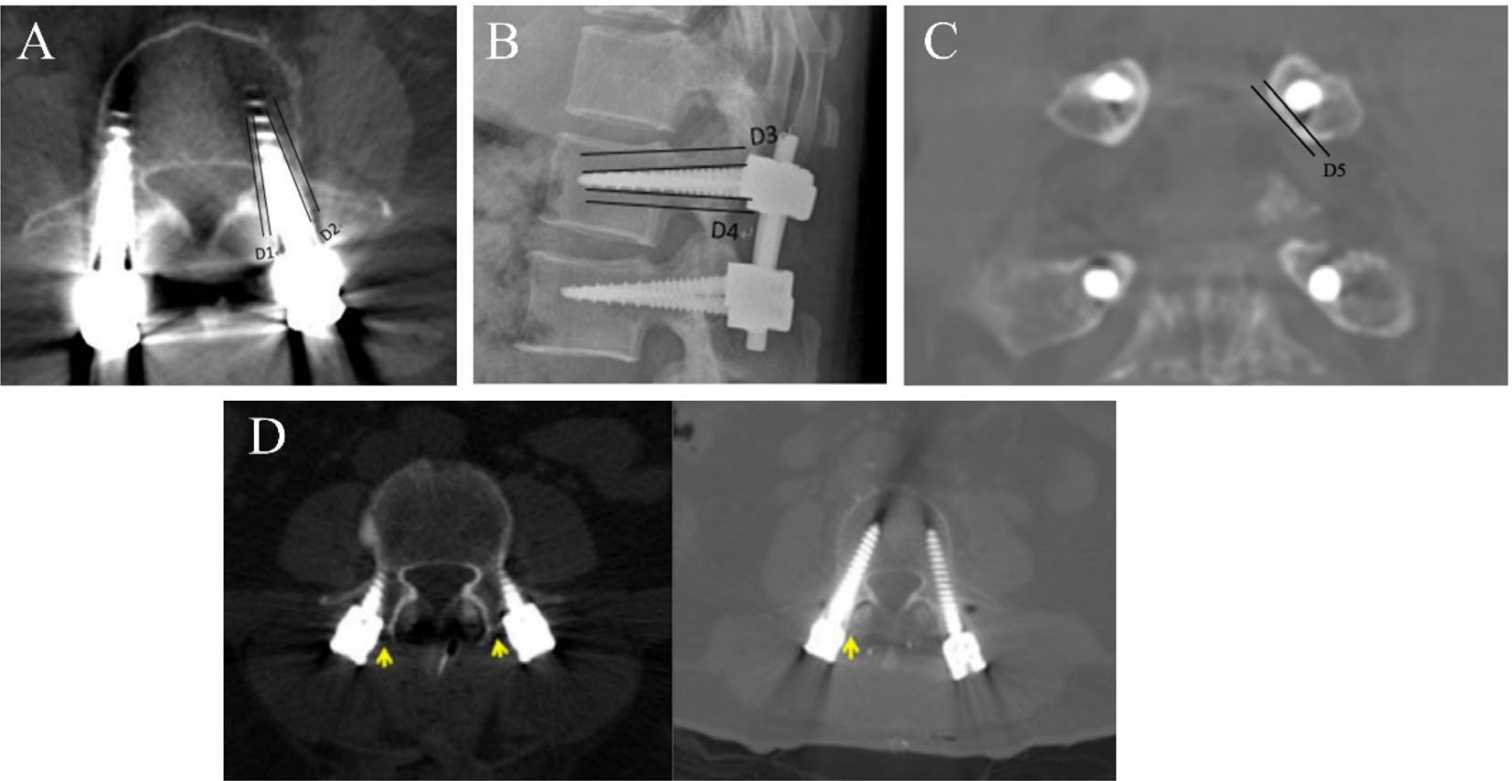
Determination criteria for pedicle screw insertion success rate: (A) Using transverse sectional images as evaluation data, when the screw does not breach the pedicle bone cortex, the transverse section with the smallest distance from the screw edge to the inner and outer cortical edges of the pedicle is selected as the measurement section. (B) Using sagittal plane images as evaluation data, when the screw does not breach the pedicle bone cortex, the sagittal section with the smallest distance from the screw edge to the head and tail cortical edges of the pedicle is selected as the measurement section. (C) Using coronal plane images as evaluation data, when the screw does not breach the pedicle bone cortex, the coronal section with the smallest distance from the screw edge to the cortical edge of the pedicle bone is selected as the measurement section. (D) When the screw does not breach the facet joint surface, the cross‐sectional image with the smallest distance from the screw edge to the adjacent facet joint surface on the lateral side is selected as the measurement section.

Cross‐sectional images were used for the evaluation. When the screw did not penetrate the cortical bone of the pedicle, the cross‐section with the smallest distance between the edge of each screw and the inner and outer cortical edges of the pedicle was selected as the measurement section (Figure 2A). In axial images used for evaluation, when the screw did not penetrate the cortical bone of the pedicle, the sagittal section with the smallest distance between the edge of each screw and the cortical edges of the pedicle on the head and tail sides was selected as the measurement section (Figure 2B). In coronal images used for evaluation, when the screw did not penetrate the cortical bone of the pedicle, the coronal section with the smallest distance between the edge of each screw and cortical edges of the pedicle was selected as the measurement section (Figure 2C). When the screw penetrated the cortical bone of the pedicle, the coronal section with the maximum distance between the edge of each screw and the cortical edges of the pedicle where penetration occurred was selected as the measurement section. The distance from the edge of the screw to the cortical bone of the pedicle was denoted as D5. When the screw was within the pedicle, D5 was recorded as a negative value; when the screw penetrated the cortical bone of the pedicle, D5 was recorded as a positive value. The unit of measurement was millimeters (mm), with the measurement being precise to 0.1 mm. Each screw was then measured and recorded. When the screw penetrated the cortical bone of the pedicle, the primary direction of penetration was recorded, that is, medial, lateral, cranial, or caudal. Each screw was measured and recorded individually, and the screw insertion excellence rate was calculated based on the total number of screws inserted during all surgeries.

Simultaneously, the degree of facet joint surface penetration on the cranial side was evaluated using the cross‐sectional images. When the screw did not penetrate the facet joint surface, the cross‐section where the distance from the edge of each screw to the adjacent facet joint surface on the cranial side was minimal and was chosen as the measurement section (Figure 2D). Before discharge, all patients underwent CT scans of the surgical segments, and DICOM 3D reconstruction software was used for image review, capturing measurement points, and measuring distances. Additionally, secondary evaluation indicators included screw insertion time, number of X‐ray fluoroscopies, surgical duration, intraoperative blood loss, AR glass usage experience, system software evaluation, and postoperative hospital stay duration.

### 3.2 Data Extraction

Population data collection, vital signs (heart rate, blood pressure, temperature, and respiration), medical history, oxygen saturation, laboratory tests (complete blood count, urine HCG, APTT, PT, TT, and FBG), electrocardiogram, and imaging examinations (CT scans) recorded the etiology of each subject undergoing surgery, along with adverse events that were collected after the informed consent form was signed. After patients signed the informed consent form, they were randomly assigned to groups. The number of spinal segments involved in each surgery, number of screws inserted, total screw placement time, amount of total fluoroscopy time, surgery duration, blood loss during surgery, equipment defects, imaging examinations (CT scans), screw placement level evaluation, facet joint breach level evaluation, postoperative hospital stay, and adverse events were recorded. A total of 150 patients who met the inclusion and exclusion criteria were randomly allocated to the experimental and control groups.

### 3.3 Statistical Analysis

For categorical data, frequency and composition ratios were used. For continuous data, mean, standard deviation, median, interquartile range, 25th and 75th percentiles, and maximum and minimum values were used for description. For baseline demographic statistics and descriptive analysis, checked for normality using Shapiro–Wilk test and for homogeneity of variances using Levene's test. Inter‐group comparisons of categorical data were conducted using the corrected chi‐square test. When more than 25% of the theoretical frequencies in the cells were less than 5, Fisher's exact probability method was used. For normally distributed continuous data, intergroup comparisons were conducted using a grouped *t*‐test. For abnormally distributed continuous data, inter‐group comparisons were performed using the Wilcoxon rank‐sum test. All the patients who used the study product were included in the analysis.

For the primary efficacy endpoint, the excellent rate of pedicle screw insertion and between‐group comparisons will be conducted using CMH chi‐square analysis adjusted for the center effect. In addition to estimating the excellent rates for the experimental and control groups, the difference in excellent rates between the groups and their 95% confidence intervals were also estimated. The methods for between‐group comparisons of other efficacy endpoints were the same as those used for baseline analysis. Within‐group (pre‐ and post‐treatment) comparisons of normally distributed continuous data will be conducted using paired *t*‐tests, whereas within‐group comparisons of abnormally distributed continuous data will be conducted using the Wilcoxon rank‐sum test.

For safety evaluation, laboratory test results will be separately described for the experimental and control groups, indicating the number and proportion of cases with normal values before treatment and abnormal values after treatment. Adverse events will be described in terms of the number of occurrences and incidence rates, with the proportion being tested using continuous corrected chi‐squared tests or Fisher's exact test. Additionally, a detailed description of all adverse events, including their manifestations, severity, and relationship to the study product, will be provided for each group. All statistical tests were conducted at a two‐sided significance level of 0.05. Statistical analysis was performed using SAS (version 9.4; SAS Institute, Cary, NC, USA).

## 4 Results

**FIGURE 3:**
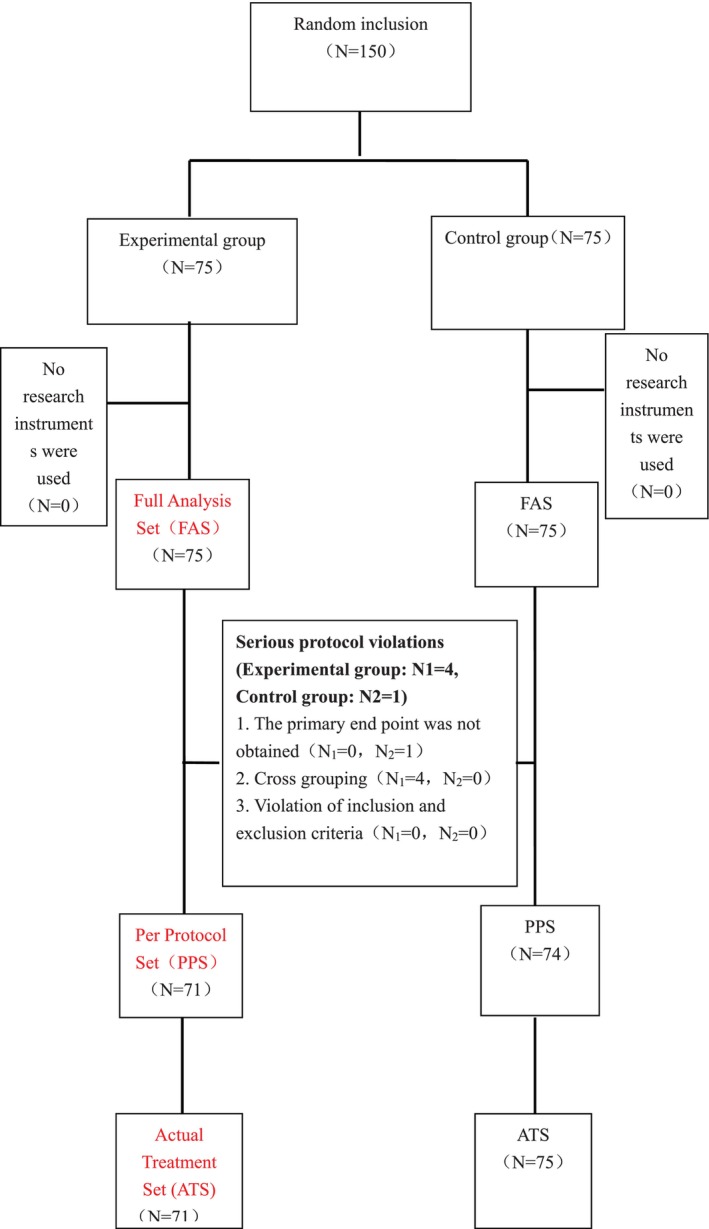
The flowchart for determining the analysis of population data sets.

This clinical study enrolled 150 subjects across three research centers, with 75 subjects randomized into the experimental group and 75 subjects in the control group. Following data review, it was determined that the FAS comprised 150 cases (75 in the experimental group and 75 in the control group), the PPS comprised 145 cases (71 in the experimental group and 74 in the control group), and the actual treatment set (ATS) comprised 146 cases (71 in the experimental group and 75 in the control group). The process used to analyze the population data sets is illustrated in Figure 3.

The patient data are summarized in Table 1. There were no statistically significant differences between the two groups in terms of average age and distribution, sex ratio, height, or average body mass index (BMI). The average ages of the experimental and control groups were 56.67 ± 11.90 and 58.79 ± 11.55 years, respectively, while the BMIs were 25.43 ± 3.51 and 25.60 ± 3.58 kg/m^2^, respectively. Additionally, multiple vital signs of the patients were analyzed, such as blood pressure, heart rate, complete blood count, and other serum tests; the results showed no significant statistical differences. The total number of pedicle screws planned for the clinical trial placement was 351 and 348 in the experimental and control groups, respectively.

**TABLE 1:** Baseline characteristics of participants.

Index	Experimental group	Control group	Statistics	*p*
Age (years)				
Number of cases (*N*)	75 (0)	75 (0)	−1.1108	0.2685
Mean ± standard deviation	56.67 ± 11.90	58.79 ± 11.55		
Median	58.65	62.49		
Q1–Q3	50.02–65.37	52.40–67.49		
Minimum value–maximum value	30.02–74.81	26.38–74.25		
Gender				
Number of cases (*N*)	75 (0)	75 (0)	0.4276	0.5132
Male	41 (54.7%)	37 (49.3%)		
Female	34 (45.3%)	38 (50.7%)		
Weight (kg)				
Number of cases (*N*)	75 (0)	75 (0)	0.5670	0.5716
Mean ± standard deviation	69.48 ± 12.21	68.45 ± 9.97		
Median	70.00	68.10		
Q1–Q3	60.00–76.80	60.00–75.00		
Minimum value–maximum value	43.80–105.00	50.00–96.00		
Height (cm)				
Number of cases (*N*)	75 (0)	75 (0)	0.9234	0.3573
Mean ± standard deviation	165.02 ± 8.38	163.66 ± 9.62		
Median	164.00	163.00		
Q1–Q3	158.00–170.00	157.00–172.00		
Minimum value–maximum value	150.00–184.50	143.00–182.00		
BMI (kg/m^2^)				
Number of cases (*N*)	75 (0)	75 (0)	−0.3006	0.7641
Mean ± standard deviation	25.43 ± 3.51	25.60 ± 3.58		
Median	25.22	24.86		
Q1–Q3	23.05–27.73	23.19–27.43		
Minimum value–maximum value	17.55–34.72	20.57–39.96		

*Note*: 1. Age = (Informed consent date − Date of birth)/365.25. 2. BMI (kg/m^2^) = Weight (kg)/Height (m^2^). 3. Experimental group: AR surgical navigation system; Control group: Conventional screw placement treatment.

### 4.1 Postoperative Follow‐Up Results

As shown in Table 2, pedicle screw placement was mostly concentrated in the segments from L1 to S1, and all patients underwent radiographic examinations. The degree of pedicle screw placement excellence is shown in Table 3. In the experimental group, 351 screws were placed, of which 344 achieved excellent placement (98.0%). In the control group, 348 screws were placed, of which 319 screws achieved excellent placement (91.7%). The proportion of clinically acceptable screws in the experimental group (98.0% excellence) was significantly higher than that in the control group (91.7%; *p* < 0.05). Furthermore, the difference in the excellence rate of screw placement (experimental group − control group) was 6.3% [3.0%–9.8%], with a *p* value of 0.003.

**TABLE 2:** Analysis results of subjects' surgeries and postoperative follow‐ups.

Index	Experimental group	Control group	Statistics	*P*
Operative segment: T9				
Number of cases (*N*)	75 (0)	75 (0)	NA	NA
Yes	0 (0.0%)	0 (0.0%)		
No	75 (100.0%)	75 (100.0%)		
Operative segment: T10				
Number of cases (*N*)	75 (0)	75 (0)	Fisher	1.0000
Yes	1 (1.3%)	0 (0.0%)		
No	74 (98.7%)	75 (100.0%)		
Operative segment: T11				
Number of cases (*N*)	75 (0)	75 (0)	Fisher	1.0000
Yes	1 (1.3%)	0 (0.0%)		
No	74 (98.7%)	75 (100.0%)		
Operative segment: T12				
Number of cases (*N*)	75 (0)	75 (0)	Fisher	1.0000
Yes	1 (1.3%)	1 (1.3%)		
No	74 (98.7%)	74 (98.7%)		
Operative segment: L1				
Number of cases (*N*)	75 (0)	75 (0)	Fisher	0.4966
Yes	0 (0.0%)	2 (2.7%)		
No	75 (100.0%)	73 (97.3%)		
Operative segment: L2				
Number of cases (*N*)	75 (0)	75 (0)	Fisher	1.0000
Yes	1 (1.3%)	2 (2.7%)		
No	74 (98.7%)	73 (97.3%)		
Operative segment: L3				
Number of cases (*N*)	75 (0)	75 (0)	0.4998	0.4796
Yes	12 (16.0%)	9 (12.0%)		
No	63 (84.0%)	66 (88.0%)		
Operative segment: L4				
Number of cases (*N*)	75 (0)	75 (0)	0.7587	0.3837
Yes	48 (64.0%)	53 (70.7%)		
No	27 (36.0%)	22 (29.3%)		
Operative segment: L5				
Number of cases (*N*)	75 (0)	75 (0)	Fisher	1.0000
Yes	72 (96.0%)	73 (97.3%)		
No	3 (4.0%)	2 (2.7%)		
Operative segment: S1				
Number of cases (N)	75 (0)	75 (0)	0.9612	0.3269
Yes	40 (53.3%)	34 (45.3%)		
No	35 (46.7%)	41 (54.7%)		
Number of operative vertebrae				
Number of cases (*N*)	75 (0)	75 (0)	Fisher	0.5717
2	49 (65.3%)	51 (68.0%)		
3	23 (30.7%)	24 (32.0%)		
1	1 (1.3%)	0 (0.0%)		
4	2 (2.7%)	0 (0.0%)		
Screw placement				
Number of cases (*N*)	75 (0)	75 (0)	Fisher	0.5169
4	49 (65.3%)	51 (68.0%)		
5	1 (1.3%)	0 (0.0%)		
6	22 (29.3%)	24 (32.0%)		
2	1 (1.3%)	0 (0.0%)		
8	2 (2.7%)	0 (0.0%)		
Name of instrument used				
Number of cases (*N*)	75 (0)	75 (0)	176.28	< 0.0001
AR surgical navigation system	71 (94.7%)	0 (0.0%)		
Conventional screw placement treatment	4 (5.3%)	75 (100.0%)		
Imaging examination				
Number of cases (*N*)	75 (0)	75 (0)	NA	NA
Yes	75 (100.0%)	75 (100.0%)		
No	0 (0.0%)	0 (0.0%)		

**TABLE 3:** (FAS) The main endpoint indicator—Analysis results of the excellent rate of screw placement (screw level, external data).

Index	Experimental group	Control group	Statistics	*p*
Screw placement excellence^#1^				
Number of cases (*N*)	351 (0)	344 (4)	11.613	0.0007
Yes	344 (98.0%)	319 (92.7%)		
No	7 (2.0%)	25 (7.3%)		
Screw placement excellence				
Number of cases (*N*)	351 (0)	348 (0)		
Yes	344 (98.0%)	319 (91.7%)		
No	7 (2.0%)	29 (8.3%)		
Difference in screw placement excellence rate (experimental group vs. control group) and 95% confidence interval^#2^		6.3% [3.0%–9.8%]		
The *p* value of the superior test		0.0003		
Difference in screw placement excellence rate (experimental group vs. control group) and 95% confidence interval^#3^		6.4% [3.0%–9.9%]		
The *p* value of the superior test		0.0003		
Difference in screw placement excellence rate (experimental group vs. control group) and 95% confidence interval^#4^		6.3% [2.9%–9.8%]		
The *p* value of the superior test		0.0003		
Difference in screw placement excellence rate (experimental group vs. control group) and 95% confidence interval^#5^		6.4% [3.0%–9.9%]		
The *p* value of the superior test		0.0003		

^#1^ For subjects who did not achieve the primary endpoint, the primary efficacy indicators were not processed and analyzed using the likelihood ratio chi‐square test or Fisher's exact test.

^#2^ For subjects who did not achieve the primary endpoint, the primary efficacy indicators were carried forward using the worst‐case imputation method and analyzed using the CMH chi‐square test adjusted for centers and the planned number of screws.

^#3^ For subjects who did not achieve the primary endpoint, the primary efficacy indicators were carried forward using the worst‐case imputation method and analyzed using the CMH chi‐square test adjusted for centers.

^#4^ For subjects who did not achieve the primary endpoint, the primary efficacy indicators were carried forward using the worst‐case imputation method and analyzed using the CMH chi‐square test adjusted for merged centers and the planned number of screws.

^#5^ For subjects who did not achieve the primary endpoint, the primary efficacy indicators were carried forward using the worst‐case imputation method and analyzed using the CMH chi‐square test adjusted for merged centers.

The analysis results of the degree of facet joint surface penetration on the lateral side after pedicle screw placement are shown in Table 4. In the experimental group, the proportions of Grade A facet joint surface penetration were 97.2%, 2.3%, 0.6%, and 0.0%, respectively. In the control group, the proportions of Grade A facet joint surface penetration were 83.3, Grade B was 7.20, and Grade C was 8.3, and 1.1%, respectively. There was a significant difference in the analysis of the degree of facet joint surface penetration on the lateral side between the two groups (*p* < 0.0001), with the experimental group demonstrating better results than the control group.

**TABLE 4:** (FAS) The secondary endpoint indicator—Analysis results of the degree of facet joint surface penetration at the head side (screw level, external data).

Index	Experimental group	Control group	Statistics	*p*
Cortical breach in the main direction of the pedicle				
Number of cases (*N*)	51 (300)	144 (204)	22.995	0.0001
Inside	15 (29.4%)	53 (36.8%)		
Outside	25 (49.0%)	24 (16.7%)		
Cephalic side	6 (11.8%)	43 (29.9%)		
Caudal	5 (9.8%)	20 (13.9%)		
ND	0 (0.0%)	4 (2.8%)		
Degree of facet joint surface penetration on the lateral side				
Number of cases (*N*)	351 (0)	348 (0)	Fisher	< 0.0001
A level	341 (97.2%)	290 (83.3%)		
B level	8 (2.3%)	25 (7.2%)		
C level	2 (0.6%)	29 (8.3%)		
ND	0 (0.0%)	4 (1.1%)		

### 4.2 Secondary Results

The results showed that the analysis of intraoperative screw placement time demonstrated an average total placement time of 16.33 ± 9.93 min in the experimental group and 30.32 ± 10.40 min in the control group. The difference in total placement time between the two groups was statistically significant (*p* < 0.05). The average time per screw placement in the experimental group was 3.51 ± 1.90 min, while in the control group it was 6.67 ± 2.35 min. The difference in the time per screw placement between the two groups was statistically significant. The average total screw placement time and average time per screw placement in the experimental group were lower than those in the control group.

The analysis of surgical time and intraoperative blood loss showed that in the experimental group, the values were 180.73 ± 47.37 min and 270.80 ± 145.81 mL, respectively, while in the control group, they were 169.89 ± 65.05 min and 293.60 ± 280.73 mL, respectively. After statistical analysis, the P‐values for surgical time were found to be 0.0201 (*p* < 0.05) and 0.8010 (*p* > 0.05) for intraoperative blood loss, indicating a significant difference in surgical time between the two groups, but no difference in intraoperative blood loss. The mean surgical time in the experimental group was longer than that in the control group.

The amount of intraoperative total fluoroscopy time showed that the average fluoroscopy time in the experimental group was 7.29 ± 2.90 times, while in the control group, it was 13.25 ± 6.02 times. There was a statistically significant difference in the amount of total fluoroscopy time between the two groups (*p* < 0.05), with fewer fluoroscopy procedures in the experimental group than in the control group (Tables S1–S7).

System software usability evaluation analysis results of AR glasses show a good user experience. User interface design, real‐time tracking of surgical instruments, and stability of software operation obtained high scores (Tables S8–S10). In addition, as shown in Figure S1, the MRI images of the control group and the experimental group showed that, in the manual group, the pedicle screws were prone to inward deviation. In summary, the AR surgical navigation system was superior to conventional manual surgery in terms of safety and effectiveness. It enhances the operability of spinal surgery, improves the accuracy and safety of screw placement, and has promising clinical prospects. It provides an effective surgical assistance tool for users, with a favorable risk–benefit ratio; it can be used to assist doctors in orthopedic spinal surgeries.

### 4.3 Follow‐Up Time and Complications

In our study, the follow‐up period was set at 2 months post‐intervention, during which participants were closely monitored for any adverse events and treatment‐related complications. We recorded the incidence of complications, including hypoalbuminemia, constipation, fever, abdominal pain, cough, vomiting, insomnia, elevated blood sugar, and bloating, in both the intervention and control groups. The results indicated an incidence of 32.0% in the experimental group compared to 26.7% in the control group, with no statistically significant difference (*p* > 0.05) (Table S11). Notably, these adverse events were determined to be unrelated to the device used. These findings provide valuable insights into the safety profile of the treatment.

## 5 Discussion

In this randomized controlled trial study, our objective was to evaluate the safety and accuracy of using the AR navigation system compared to the conventional manual placement of pedicle screws in 150 patients. It demonstrated significant improvements in the effectiveness and safety of pedicle screw placement using an AR navigation system. In the AR group, 98% of pedicle screw trajectories were satisfactory, whereas in the manual group, 92.7% of trajectories were satisfactory, with statistical significance between the two. Importantly, the number of clinically acceptable screw insertions was significantly higher in the AR group than that in the traditional group. This aligns with similar findings reported in previous studies, such as the Nils study, where cone beam CT (CBCT) was used, similar to the navigation technology used in our experiment but with a lower accuracy rate of 89.0%.

### 5.1 Clinical Pain Points

In spinal surgery, it is crucial to correctly and appropriately place pedicle screws and other instruments to ensure spinal integrity. In theory, the use of robots and navigation systems to assist in the placement of pedicle screws ensures that they follow the planned trajectory and improves the accuracy of the surgery. The application of robots and navigation systems in spinal surgery is not a novel concept.

For example, multiple studies show that, although the Spine Assist/Renaissance robot can improve surgical accuracy and reduce radiation exposure, there is a significant variability in the placement of pedicle screws. According to the Gertzbin–Robbins classification, the clinical acceptability of pedicle screw placement using CT‐guided traditional manual techniques is higher than that using the Spine Assist/Renaissance robot [7, 25]. Meanwhile, a prospective study suggests that there is no significant difference in the accuracy of pedicle screw placement between robot‐assisted and traditional open surgeries [26]. TiRobot is currently receiving considerable attention due to its ability to track a patient's full‐body and respiratory movements in real time [7]. Wei et al. contend that TiRobot demonstrates a superior perfect placement rate (Grade A) compared with manual insertion in spinal surgery [27].

However, despite the notable improvements in screw placement brought about by surgical robots compared with traditional techniques, cost‐effectiveness remains a significant limiting factor for the widespread clinical application of orthopedic robots.

### 5.2 AR Outcomes and Advantages

We propose the use of AR guidance in addition to traditional manual screw insertion to assist clinicians in achieving more accurate screw placement. AR surgical navigation systems have been developed and produced on the basis of AR technology. The AR navigation system processes, analyses, integrates, and outputs the preoperative imaging data of patients into the workstation, generating personalized, objective, and precise 3D simulated images to ensure high conformity between reconstructed 3D images and actual anatomical structures during surgery. Using registration techniques to precisely match the coordinates of virtual and real objects and present them to the surgeon, the system provides high image clarity, resolution, structured views, and strong stereoscopic perception. During surgery, it assists surgeons in the real time, rapid, and accurate identification of anatomical structures and their positional relationships, enabling precise surgical operations.

### 5.3 Clinical Outcomes and Advantages

The experimental results indicate that the number of pedicle cortical violations was higher in the manual group than in the navigation group, with 144 cases (70.6%) and 51 cases (17%), respectively. Furthermore, the predominant direction of violation in the traditional surgery group was medial (36.8%). Fortunately, none of the patients required revision surgeries. The reasons for both groups experiencing pedicle cortical violations are attributable to neither group deviating from manual screw placement techniques. This was also observed in Nils' CBCT group study [28]. However, these data may also be influenced by the clinical experience of operating physicians in hospitals. In addition, the directionality of pedicle screw violations in clinical practice may lead to revision surgeries which affect patient recovery time, among other factors.

The degree of facet joint surface penetration was remarkably superior in the navigation group. Compared to the 83.3% A‐level penetration in the manual group, the navigation group's 97.2% A‐level penetration was statistically significant. The navigation group had significantly longer surgical times than those of the manual group. The results, which show a significant reduction in overall surgical time compared with manual techniques, differ somewhat from those reported in previous studies by Ryan and Khanna [29]. We believe that this discrepancy may be partly attributable to the initial setup and preparation of the navigation equipment. However, in terms of total screw placement time during surgery, the navigation group (mean: 16.33) was significantly shorter than the traditional group (mean: 30.32), with a significant statistical difference between the two.

### 5.4 Limitations and Strengths

While previous research has shown the integration of various navigation technologies with spinal surgery, we are the first to conduct a randomized controlled trial comparing the accuracy and safety of AR‐based virtual AR navigation‐guided surgery with manual pedicle screw placement and without AR assistance.

Unfortunately, despite our efforts to control for biases through various measures, the clinical experience of the surgeons remains a confounding factor that is difficult for us to control. Although this study, along with other research findings [30, 31, 32], demonstrates the significant advantages of the AR navigation system in spinal surgery, we also acknowledge that there are some limitations to our study. First, although the sample size is sufficient for statistical analysis, further validation is needed in large‐scale clinical applications. Second, this study focuses on the lumbar spine, lacking experimental data on the thoracic and cervical regions, which is a major limitation of our current research. In the future, we plan to conduct further randomized controlled trials to explore the application of AR navigation in other spinal surgery scenarios, thereby expanding its indications.

## 6 Conclusion

In this paper, we present an AR surgical navigation system that outperforms conventional manual surgery, exhibiting excellent safety and efficacy. This system enhances the operability of spinal procedures, increasing the accuracy and safety of screw placement while demonstrating promising prospects for clinical application. By offering an effective surgical assistance tool, it presents a favorable risk–benefit ratio, making it a valuable resource for doctors performing orthopedic spinal surgeries.

## Author Contributions

Yichao Ma and Jiangpeng Wu contributed equally to this work and should be regarded as co‐first authors. Yichao Ma: Data collection, data analysis, and essay writing. Jiangpeng Wu and Yanlong Dong: Data collection and essay revision. Yichao Ma and Jiangpeng Wu followed the patients and coordinated the study. Yichao Ma and Yanlong Dong analyzed and critically discussed the results of this paper. Xiaojun Ma and Hongmei Tang: Critical revision and discussion of the final version of the manuscript. All authors have read and approved the final manuscript.

## Ethics Statement

The study was approved by the Ethics Committee of Shanghai General Hospital and conformed to the provisions of the Declaration of Helsinki.

## Consent

All participants provided the written informed consent.

## Conflicts of Interest

The authors declare no conflicts of interest.

## Generative AI in Scientific Writing

The AI technique was applied to improve the readability and language under the supervision of corresponding author.

## Supporting information


**Data S1.** Supporting Information.

## Data Availability

The data sets generated and/or analyzed during the current study are available from the corresponding author upon reasonable request.
