# Supplementary material for: Augmented Reality Navigation System Enhances the Accuracy of Spinal Surgery Pedicle Screw Placement: A Randomized, Multicenter, Parallel‐Controlled Clinical Trial
Source: Orthop Surg. 2025 Jan 15;17(2):631–43. doi: 10.1111/os.14295 (PMC11787979; doi:10.1111/os.14295)
Supplement: Supplementary file 1 — Data S1. Supporting Information. [file OS-17-631-s001.docx]

----------------------------------------------------------------------------------------------------------------------

Index Experimental group Control group Statistics P-value

----------------------------------------------------------------------------------------------------------------------

Systolic pressure（mmHg）

Number of cases（N） 75 ( 0) 75 ( 0) -1.6595 0.0991

Mean ± standard deviation 132.73 ±20.69 138.08 ±18.72

Median 131.00 140.00

Q1;Q3 119.00 ;143.00 126.00 ;151.00

Minimum value; Maximum value 96.00 ;215.00 93.00 ;182.00

Diastolic blood pressure（mmHg）

Number of cases（N） 75 ( 0) 75 ( 0) -0.3457 0.7300

Mean ± standard deviation 80.91 ±10.70 81.55 ±11.94

Median 81.00 80.00

Q1;Q3 74.00 ;88.00 74.00 ;90.00

Minimum value; Maximum value 55.00 ;104.00 55.00 ;110.00

Heart rate (times/min)

Number of cases（N） 75 ( 0) 75 ( 0) 0.7646 0.4457

Mean ± standard deviation 82.01 ±13.00 80.47 ±11.74

Median 80.00 79.00

Q1;Q3 72.00 ;89.00 72.00 ;89.00

Minimum value; Maximum value 53.00 ;120.00 57.00 ;121.00

Body temperature (℃)

Number of cases（N） 75 ( 0) 75 ( 0) -0.4364 0.6632

Mean ± standard deviation 36.47 ±0.29 36.49 ±0.27

Median 36.50 36.50

Q1;Q3 36.30 ;36.60 36.30 ;36.60

Minimum value; Maximum value 36.00 ;37.20 36.00 ;37.20

Breathing (cycles per minute)

Number of cases（N） 75 ( 0) 75 ( 0) 2.3185 0.0218

Mean ± standard deviation 18.23 ±0.99 17.87 ±0.91

Median 18.00 18.00

Q1;Q3 18.00 ;19.00 18.00 ;18.00

Minimum value; Maximum value 16.00 ;20.00 16.00 ;20.00

----------------------------------------------------------------------------------------------------------------------

**Supplementary Table1: Results of Vital Signs Analysis during Subject Screening Period**

----------------------------------------------------------------------------------------------------------------------

Index Experimental group Control group Statistics P-value

---------------------------------------------------------------------------------------------------------------------- Urine HCG

Number of cases（N） 34 ( 41) 38 ( 37) Fisher 0.6368

Negative 21 ( 61.8%) 21 ( 55.3%)

Positive 0 ( 0.0%) 0 ( 0.0%)

Not done 0 ( 0.0%) 0 ( 0.0%)

Not applicable (postmenopausal or sterilized women) 13 ( 38.2%) 17 ( 44.7%)

----------------------------------------------------------------------------------------------------------------------

**Supplementary Table 2: Analysis Results of Urine hCG during Subject Screening Period**

----------------------------------------------------------------------------------------------------------------------

Index Experimental group Control group Statistics P-value

----------------------------------------------------------------------------------------------------------------------

Blood oxygen saturation examination

Number of cases（N） 75 ( 0) 75 ( 0) Fisher 1.0000

YES 74 ( 98.7%) 75 (100.0%)

NO 1 ( 1.3%) 0 ( 0.0%)

Blood oxygen saturation (%)

Number of cases（N） 74 ( 1) 75 ( 0) 0.3026 0.7626

Mean ± standard deviation 97.28 ±1.88 97.20 ±1.40

Median 98.00 97.00

Q1;Q3 97.00 ;99.00 96.00 ;98.00

Minimum value; Maximum value 91.00 ;100.00 92.00 ;100.00

----------------------------------------------------------------------------------------------------------------------

**Supplementary Table 3: Analysis Results of Blood Oxygen Saturation Examination During Subject Screening**

---------------------------------------------------------------------------------------------------------------------

Index Experimental group Control group Statistics P-value

----------------------------------------------------------------------------------------------------------------------

Complete blood count

Number of cases（N） 75 ( 0) 75 ( 0) NA NA

YES 75 (100.0%) 75 (100.0%)

NO 0 ( 0.0%) 0 ( 0.0%)

Platelet count

Number of cases（N） 75 ( 0) 75 ( 0) Fisher 0.5330

Normal 71 ( 94.7%) 68 ( 90.7%)

Abnormalities clinically insignificant 4 ( 5.3%) 6 ( 8.0%)

Abnormal with clinical significance 0 ( 0.0%) 1 ( 1.3%)

Coagulation Function Tests

Number of cases（N） 75 ( 0) 75 ( 0) NA NA

YES 75 (100.0%) 75 (100.0%)

NO 0 ( 0.0%) 0 ( 0.0%)

Activated Partial Thromboplastin Time (APTT)

Number of cases（N） 75 ( 0) 75 ( 0) Fisher 0.8344

Normal 60 ( 80.0%) 62 ( 82.7%)

Abnormalities clinically insignificant 15 ( 20.0%) 13 ( 17.3%)

Abnormal with clinical significance 0 ( 0.0%) 0 ( 0.0%)

Not tested 0 ( 0.0%) 0 ( 0.0%)

Prothrombin Time (PT)

Number of cases（N） 75 ( 0) 75 ( 0) Fisher 1.0000

Normal 70 ( 93.3%) 71 ( 94.7%)

Abnormalities clinically insignificant 5 ( 6.7%) 4 ( 5.3%)

Abnormal with clinical significance 0 ( 0.0%) 0 ( 0.0%)

Not tested 0 ( 0.0%) 0 ( 0.0%)

Thrombin Time (TT)

Number of cases（N） 75 ( 0) 75 ( 0) Fisher 1.0000

Normal 73 ( 97.3%) 73 ( 97.3%)

Abnormalities clinically insignificant 2 ( 2.7%) 2 ( 2.7%)

Abnormal with clinical significance 0 ( 0.0%) 0 ( 0.0%)

Not tested 0 ( 0.0%) 0 ( 0.0%)

---------------------------------------------------------------------------------------------------------------------

**Supplementary Table 4: Analysis Results of Blood Routine and Coagulation Function Tests During Subject Screening Period**

---------------------------------------------------------------------------------------------------------------------

Index Experimental group Control group Statistics P-value

-------------------------------------------------------------------------------------------------------------------- Total Screw Placement Time (min)

Number of cases（N） 75 ( 0) 75 ( 0) -8.4275 <.0001

Mean ± standard deviation 16.33 ±9.93 30.32 ±10.40

Median 14.00 30.00

Q1;Q3 12.00 ;20.00 23.00 ;35.00

Minimum value; Maximum value 6.00 ;88.00 10.00 ;67.00

Average Time per Screw Placement (min)

Number of cases（N） 75 ( 0) 75 ( 0) -9.0887 <.0001

Mean ± standard deviation 3.51 ±1.90 6.67 ±2.35

Median 3.30 6.00

Q1;Q3 2.80 ;3.80 5.00 ;7.50

Minimum value; Maximum value 1.70 ;17.60 2.50 ;14.30

----------------------------------------------------------------------------------------------------------------------

**Supplementary Table 5 (FAS) Secondary Endpoint - Screw Placement Time Analysis Results**

----------------------------------------------------------------------------------------------------------------------

Index Experimental group Control group Statistics P-value

----------------------------------------------------------------------------------------------------------------------

Number of X-ray Fluoroscopy Shots

Number of cases（N） 75 ( 0) 75 ( 0) -7.7225 <.0001

Mean ± standard deviation 7.29 ±2.90 13.25 ±6.02

Median 6.00 11.00

Q1;Q3 6.00 ;8.00 9.00 ;16.00

Minimum value; Maximum value 4.00 ;16.00 5.00 ;42.00

----------------------------------------------------------------------------------------------------------------------

**Supplementary Table 6 (FAS) Secondary Endpoint - Analysis of X-ray Fluoroscopy Shots**

----------------------------------------------------------------------------------------------------------------------

Index Experimental group Control group Statistics P-value

---------------------------------------------------------------------------------------------------------------------- Surgical Time (min)

Number of cases（N） 75 ( 0) 75 ( 0) 2.3244 0.0201

Mean ± standard deviation 180.73 ±47.37 169.89 ±65.05

Median 180.00 150.00

Q1;Q3 150.00 ;210.00 135.00 ;185.00

Minimum value; Maximum value 93.00 ;300.00 82.00 ;520.00

Intraoperative Blood Loss (ml)

Number of cases（N） 75 ( 0) 75 ( 0) 0.2520 0.8010

Mean ± standard deviation 270.80 ±145.81 293.60 ±280.73

Median 200.00 200.00

Q1;Q3 200.00 ;300.00 200.00 ;300.00

Minimum value; Maximum value 30.00 ;800.00 20.00 ;2200.0

----------------------------------------------------------------------------------------------------------------------

**Supplementary Table 7 (FAS) Secondary Endpoint Analysis Results - Surgical Time and Intraoperative Blood Loss**

----------------------------------------------------------------------------------------------------------------------

Index Experimental group Control group Statistics P-value

----------------------------------------------------------------------------------------------------------------------

Wear ability Score (points)

Number of cases（N） 71 ( 4) 0 ( 75) NA NA

Mean ± standard deviation 3.83 ±0.38 NA ±NA

Median 4.00 NA

Q1;Q3 4.00 ;4.00 NA ;NA

Minimum value; Maximum value 3.00 ;4.00 NA ;NA

Wear ability Score

Number of cases（N） 71 ( 4) 0 ( 75) NA NA

0 Score 0 ( 0.0%) 0 ( 0.0%)

1 Score 0 ( 0.0%) 0 ( 0.0%)

2 Score 0 ( 0.0%) 0 ( 0.0%)

3 Score 12 ( 16.9%) 0 ( 0.0%)

4 Score 59 ( 83.1%) 0 ( 0.0%)

Image Quality Score (points)

Number of cases（N） 71 ( 4) 0 ( 75) NA NA

Mean ± standard deviation 3.89 ±0.36 NA ±NA

Median 4.00 NA

Q1;Q3 4.00 ;4.00 NA ;NA

Minimum value; Maximum value 2.00 ;4.00 NA ;NA

Image Quality Score

Number of cases（N） 71 ( 4) 0 ( 75) NA NA

0 Score 0 ( 0.0%) 0 ( 0.0%)

1 Score 0 ( 0.0%) 0 ( 0.0%)

2 Score 1 ( 1.4%) 0 ( 0.0%)

3 Score 6 ( 8.5%) 0 ( 0.0%)

4 Score 64 ( 90.1%) 0 ( 0.0%)

Interaction Score(points)

Number of cases（N） 71 ( 4) 0 ( 75) NA NA

Mean ± standard deviation 3.83 ±0.38 NA ±NA

Median 4.00 NA

Q1;Q3 4.00 ;4.00 NA ;NA

Minimum value; Maximum value 3.00 ;4.00 NA; NA

----------------------------------------------------------------------------------------------------------------------

**Supplementary Table 8 (FAS) Secondary Endpoint - Analysis Results of AR Glasses Usage Experience**

---------------------------------------------------------------------------------------------------------------------- Index Experimental group Control group Statistics P-value

----------------------------------------------------------------------------------------------------------------------

Interaction Score

Number of cases（N） 71 ( 4) 0 ( 75) NA NA

0 Score 0 ( 0.0%) 0 ( 0.0%)

1 Score 0 ( 0.0%) 0 ( 0.0%)

2 Score 0 ( 0.0%) 0 ( 0.0%)

3 Score 12 ( 16.9%) 0 ( 0.0%)

4 Score 59 ( 83.1%) 0 ( 0.0%)

Use total score (points)

Number of cases（N） 71 ( 4) 0 ( 75) NA NA

Mean ± standard deviation 11.55 ±0.86 NA ±NA

Median 12.00 NA

Q1;Q3 11.00 ;12.00 NA ;NA

Minimum value; Maximum value 8.00 ;12.00 NA ;NA

AR glasses experience compliance rate (≥8 points)

Number of cases（N） 71 ( 4) 0 ( 75) NA NA

YES 71 (100.0%) 0 ( 0.0%)

NO 0 ( 0.0%) 0 ( 0.0%)

----------------------------------------------------------------------------------------------------------------------

**Supplementary Table 9 (FAS) Secondary Endpoint - Analysis Results of AR Glasses Usage Experience** **(continued)**

----------------------------------------------------------------------------------------------------------------------

Index Experimental group Control group Statistics P-value

----------------------------------------------------------------------------------------------------------------------

User Interface Design

Number of cases（N） 71 ( 4) 0 ( 75) NA NA

Mean ± standard deviation 1.96 ±0.20 NA ±NA

Median 2.00 NA

Q1;Q3 2.00 ;2.00 NA ;NA

Minimum value; Maximum value 1.00 ;2.00 NA ;NA

User Interface Design

Number of cases（N） 71 ( 4) 0 ( 75) NA NA

0 Score 0 ( 0.0%) 0 ( 0.0%)

1 Score 3 ( 4.2%) 0 ( 0.0%)

2 Score 68 ( 95.8%) 0 ( 0.0%)

Real-time Tracking of Surgical Instruments

Number of cases（N） 71 ( 4) 0 ( 75) NA NA

Mean ± standard deviation 1.90 ±0.30 NA ±NA

Median 2.00 NA

Q1;Q3 2.00 ;2.00 NA ;NA

Minimum value; Maximum value 1.00 ;2.00 NA ;NA

Real-time Tracking of Surgical Instruments

Number of cases（N） 71 ( 4) 0 ( 75) NA NA

0 Score 0 ( 0.0%) 0 ( 0.0%)

1 Score 7 ( 9.9%) 0 ( 0.0%)

2 Score 64 ( 90.1%) 0 ( 0.0%)

Stability of Software Operation

Number of cases（N） 71 ( 4) 0 ( 75) NA NA

Mean ± standard deviation 1.96 ±0.20 NA ±NA

Median 2.00 NA

Q1;Q3 2.00 ;2.00 NA ;NA

Minimum value; Maximum value 1.00 ;2.00 NA ;NA

---------------------------------------------------------------------------------------------------------------------

**Supplementary Table 10 Secondary Endpoint - System Software Usability Evaluation Analysis Results**

----------------------------------------------------------------------------------------------------------------------

Index Experimental group Control group Statistics P-value

----------------------------------------------------------------------------------------------------------------------

Adverse events

Number of cases（N） 75 ( 0) 75 ( 0) 0.5151 0.4729

Yes 24 ( 32.0%) 20 ( 26.7%)

No 51 ( 68.0%) 55( 73.3%)

----------------------------------------------------------------------------------------------------------------------

**Supplementary Table 11 Safety evaluation indicators (FAS) -Adverse events Results**


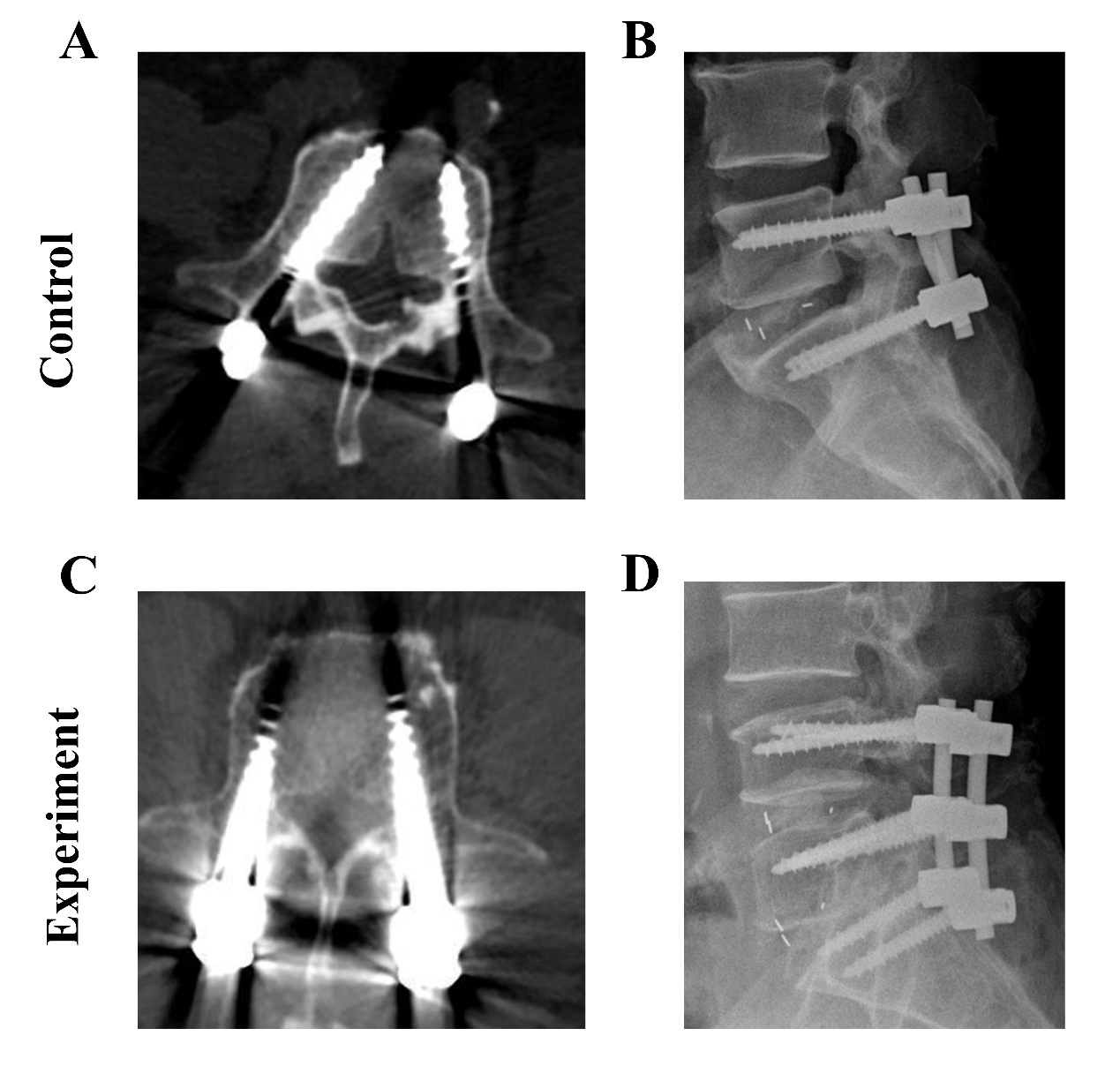


**Supplementary Figure 1** The MRI images of the control group and the experimental group.

A. Preoperative lumbar spine MRI of the control group patients. B. Postoperative sagittal plane X-ray of the control group patients. C. Preoperative lumbar spine MRI of the experimental group patients. D. Postoperative sagittal plane X-ray of the experimental group patients.
